# Childcare Service Centers’ Preferences and Intentions to Use a Web-Based Program to Implement Healthy Eating and Physical Activity Policies and Practices: A Cross-Sectional Study

**DOI:** 10.2196/jmir.3639

**Published:** 2015-04-30

**Authors:** Sze Lin Yoong, Christopher Michael Williams, Meghan Finch, Rebecca Wyse, Jannah Jones, Megan Freund, John Henry Wiggers, Nicole Nathan, Pennie Dodds, Luke Wolfenden

**Affiliations:** ^1^Hunter New England Local Health DistrictHunter New England Population HealthWallsendAustralia; ^2^The University of NewcastleSchool of Medicine and Public HealthCallaghanAustralia; ^3^The George Institute for Global HealthSydneyAustralia

**Keywords:** obesity, long day care centers, childcare centers, guideline adherence

## Abstract

**Background:**

Overweight and obesity is a significant public health problem that impacts a large number of children globally. Supporting childcare centers to deliver healthy eating and physical activity-promoting policies and practices is a recommended strategy for obesity prevention, given that such services provide access to a substantial proportion of children during a key developmental period. Electronic Web-based interventions represent a novel way to support childcare service providers to implement such policies and practices.

**Objective:**

This study aimed to assess: (1) childcare centers’ current use of technology, (2) factors associated with intention to use electronic Web-based interventions, and (3) Web-based features that managers rated as useful to support staff with implementing healthy eating and physical activity-promoting policies and practices.

**Methods:**

A computer-assisted telephone interview (CATI) was conducted with service managers from long day care centers and preschools. The CATI assessed the following: (1) childcare center characteristics, (2) childcare centers’ use of electronic devices, (3) intention to use a hypothetical electronic Web-based program—assessed using the Technology Acceptance Model (TAM) with ratings between 1 (strongly disagree) and 7 (strongly agree), and (4) features rated as useful to include in a Web-based program.

**Results:**

Overall, 214 service centers out of 277 (77.3%) consented to participate. All service centers except 2 reported using computers (212/214, 99.1%), whereas 40.2% (86/214) used portable tablets. A total of 71.9% (151/210) of childcare service managers reported a score of 6 or more for intention to use a hypothetical electronic Web-based program. In a multivariable logistic regression analysis, intention to use the program was significantly associated with perceived ease of use (*P*=.002, odds ratio [OR] 3.9, 95% CI 1.6-9.2) and perceived usefulness (*P*<.001, OR 28,95% CI 8.0-95.2). Features reported by service managers as useful or very useful for a Web-based program included decision-support tools to support staff with menu planning (117/129, 90.7%), links to relevant resources (212/212, 100%), updated information on guidelines (208/212, 98.1%), and feedback regarding childcare center performance in relation to other childcare centers (212/212, 100%).

**Conclusions:**

Childcare service managers reported high intention to use a Web-based program and identified several useful features to support staff to implement healthy eating and physical activity policies and practices. Further descriptive and intervention research examining the development and use of such a program to support childcare centers with the implementation of healthy eating and physical activity-promoting policies and practices is warranted.

## Introduction

Overweight and obesity adversely impacts a large proportion of the population globally, accounting for at least 2.8 million deaths annually and 35.8 million disability-adjusted life years [1]. It is estimated that approximately 60% of adults in developing countries are overweight or obese [2]. Globally, approximately 43 million preschool-aged children were overweight or obese in 2010, with this figure projected to reach 60 million by 2020 [3]. Children who are overweight or obese are up to ten times more likely to develop non-insulin-dependent diabetes and eight times more likely to develop cardiovascular disease during childhood [4]. Overweight or obese children also have a significantly increased risk of adult morbidity and up to three times increased risk of adult mortality as compared to children within the healthy weight range [4]. As a consequence, interventions to reduce childhood overweight and obesity are recommended to reduce the risk of chronic disease in both childhood and adulthood [5,6].

Center-based childcare services represent a promising setting for obesity prevention interventions targeting young children, with between 60% and 80% of young children in countries including Australia and the United States attending these centers [7,8]. Recognizing this opportunity, guidelines for childcare centers recommend the implementation of healthy eating and physical activity-promoting policies and practices [9,10]. Findings from reviews of randomized and quasi-experimental trials suggest that the implementation of a number of policies and practices in childcare, including providing programmed time for physical activity and improving nutritional quality of food provided, are effective in improving child diet and physical activity levels, and preventing unhealthy weight gain [5,11,12].

Although research supports the implementation of healthy eating and physical activity policies and practices in childcare service settings [9,10], their adoption by childcare centers is suboptimal. A study conducted in 20 childcare centers in the United States found that approximately 30% of childcare centers met guideline recommendations for the provision of fruit and vegetables [13]. Further, in a sample of 96 childcare centers in the United States, only 14% of childcare centers provided 120 minutes of active play per day and 40% provided two or more occasions of teacher-led physical activity [14]. Similarly, a study of 261 Australian childcare centers reported that only 41% of preschools and 48% of long day care centers had a written physical activity policy, and between 46% and 60% undertook daily, programmed, fundamental movement skills programs for children aged 2 to 3 years and 3 to 5 years [15].

A small number of trials have been conducted to improve the implementation of healthy eating and physical activity-promoting practices in the childcare setting [16,17]. Interventions which included multiple organizational change strategies, such as the provision of regular face-to-face or telephone support by qualified health staff and the provision of feedback and resources, have been shown to be effective [17-19]. However, such interventions are often resource intensive, and expensive to deliver to large numbers of centers. Most previous trials have also been conducted on a small number of childcare centers (ie, less than 30) [16,20,21], providing limited information regarding the effectiveness of such interventions when scaled up and delivered to all eligible centers [22].

Web-based interventions, including the provision of online training and resources, and interactive tools, represent a promising way of providing population-wide support to childcare centers. Such interventions enable the provision of support to large numbers of childcare centers at a fraction of the cost of other modalities. Further, childcare centers report having existing computing infrastructure and are familiar with the use of Web-based technology, thus increasing the likelihood of engaging in such interventions [8,23,24]. Research examining the effectiveness of such electronic interventions in facilitating the implementation of health-promoting policies and practices in community-based settings is scarce, with an updated Agency for Healthcare and Research Quality review failing to identify such interventions in the childcare setting [25]. Research from primary care and hospital settings, however, demonstrate that electronic interventions can be used to improve clinicians’ practices through decision-support tools, performance monitoring and feedback, information communication and prompts, and reminder functions [26,27].

Despite the promise of using electronic modalities, the public health impacts of such interventions are often impeded by low uptake and dropout or attrition in use [28,29]. Frequently reported barriers to uptake include the lack of access to appropriate infrastructure, setup costs, limited integration with existing operating electronic systems, lack of considered implementation, and failure of interventions to meet the immediate needs of users (eg, program being too complex and including features not acceptable or relevant to end users [13,30]) [29,31]. Recognizing such challenges, theories such as the Technology Acceptance Model (TAM) recommend that formative examination of factors associated with end-user intention to use a new electronic system be conducted to maximize the likelihood of end-user adoption [32,33]. Further, assessment of users’ preferences regarding the content and type of features they would like available in a Web-based intervention is likely to facilitate uptake and ongoing user engagement [13,34].

To provide relevant information to guide the design and implementation of a Web-based intervention for childcare centers, a survey was conducted with childcare center managers to (1) identify centers’ access to the Web and Web-access devices, and (2) identify factors associated with managers’ intention to use a Web-based program designed to support implementation of healthy eating and physical activity-promoting policies and practices. Further, the study examined managers’ preferences for features to include in a Web-based program to support the implementation of such policies and practices in childcare centers.

## Methods

### Ethics Approval

Ethics approval was obtained from Hunter New England Local Health District (HNELHD) Human Research Ethics Committee (06/07/26/4.04) and the University of Newcastle (H-2008-0341).

### Design and Setting

A cross-sectional study was conducted in the state of New South Wales (NSW), Australia. There were approximately 566,862 children aged between 0 and 5 years [35], and 2587 childcare centers—preschools and long day care centers—in the state [36].

### Sample

Childcare centers, including preschools and long day care centers, in NSW provide education and center-based care for children aged 0 to 5 years. Childcare centers in NSW were identified from a list of all licensed centers supplied by the regulator—The State Office of Childcare. Using the RAND function in Microsoft Excel, a random sample of 277 out of 2587 (10.71%) eligible childcare centers within NSW were selected to participate in the study. Childcare centers that catered exclusively to children with special needs or that operated within a primary school were ineligible for participation. Childcare centers located within a particular region of the state were also excluded due to their participation in a separate implementation trial [37]. Licensing and accreditation requirements regarding healthy eating and physical activity are identical for both preschools and long day care centers [38]. As such, preferences for Web-based features to support implementation of obesity prevention policies and practices were not examined separately for the two types of childcare centers. In Australia, all childcare centers that provide government-subsidized childcare benefits are required by federal legislation to use a government-mandated, Web-based Child Care Management System (CCMS) to log enrolments, store essential service information, and enable calculation of childcare benefits [24]. All centers, regardless of whether they had access to CCMS software, were eligible to participate in this study.

### Recruitment and Data Collection

Service managers of all selected childcare centers were sent an information letter inviting them to participate in the study survey, which was conducted from October to December 2013. Up to 2 weeks following the mailing of the invitations, research assistants telephoned the service managers to confirm eligibility and to gain consent. If a service manager was unavailable to complete the survey, an alternative staff member was nominated to participate in the survey. Data was collected via a 25-minute computer-assisted telephone interview (CATI).

### Measures

#### Service Characteristics

Service managers were asked to report the following: the center operating times, number of children enrolled, number of educators (ie, carers with primary contact role), and whether they were a childcare benefit-approved center using CCMS to report child enrolment details.

#### Access and Use of Internet and Associated Equipment

Service managers were asked to report whether they had access to the Internet, and whether they used computers and tablets in their center. If such infrastructure existed, service managers were asked to identify the purposes for using such infrastructure including the following: administrative tasks, completion of reporting requirements, staff education, child education, searching for information, accessing emails, recording and planning of daily program and activities, reporting progress to parents, taking photographs of children’s activities, and other purposes.

#### Factors Associated With Intention to Use a Web-Based Program to Implement Policies and Practices

Items from the Technology Acceptance Model were used to assess intention to use the hypothetical electronic Web-based program. TAM is one of the most parsimonious models assessing end-user intentions to adopt a new information technology system [39]. This model posits that attitudinal characteristics of end users, including perceived usefulness and perceived ease of use, are predictors of intentions to adopt new information technology systems. Reviews of empirical studies report that TAM accounts for between 40% and 70% of variance in explaining intention to use new electronic systems in health care, university, and commercial work settings [33,39,40]. Such studies report high internal consistency for each item (ie, Cronbach alpha >.8 for each item) [39]. This model has also shown high validity when used in countries external to Northern America, where originally developed [41], including China [42] and Switzerland [43]. Further, systematic reviews also report positive associations between behavioral intention to use and TAM constructs, with actual use of program [44,45]. Specifically, systematic reviews examining the relationship between behavioral intentions—as measured by TAM—and actual use of technology found a positive association for approximately 90% of included studies [44,45].

Similar to other studies utilizing TAM [46], service managers were asked to rate on a 7-point scale—1 (strongly disagree) to 7 (strongly agree)—the perceived usefulness, perceived ease of use, and intention to use an electronic online system to support implementation of healthy eating and physical activity policies and practices. The questionnaire was pilot-tested with 6 childcare center managers and, as a result, some minor modifications to the wording of the questions were made to increase relevance to the setting. Perceived usefulness (ie, the perception that using this system will help users achieve gains in job performance) [41] was assessed by the following statements: It would be useful to *assist staff*, *improve staff performance*, *increase staff productivity*, and *enhance effectiveness of staff delivery of healthy eating and physical activity-promoting policies and practices*. Perceived ease of use (ie, the degree of ease associated with using a system) [41] examined the following: *ease of interaction with the program*, *mental effort required*, *ease of use of program*, and *ease to get the program to do what they wanted it to do*. Intention to use were assessed by asking managers whether they: *intended to use the system*, *predicted they would use the system*, and *planned to use such a system if it were made available to them* (see Multimedia Appendix 1 for questionnaire).

#### Features to Support Healthy Eating and Physical Activity Policy and Practice Implementation

Center managers were asked to rate on a 4-point scale—1 (very unhelpful) to 4 (very helpful)—whether they perceived the following features as helpful to support staff implement healthy eating and physical activity-promoting policies and practices: *interaction and communication tools*, including (1) chat rooms, (2) discussion boards, and (3) email feedback or phone support from health care service staff, *provision of educational materials* (ie, lunchbox or menu-planning ideas, physical activity ideas, links to other helpful websites), *decision-support systems* (ie, menu-planning tool), *performance feedback and monitoring tools* (ie, features to monitor progress over time and in relation to other services, and tools to help staff with prioritizing tasks), and *prompts and reminders* [18]. Such questions were based on consultations with childcare center managers, health promotion practitioners who support services to implement such practices, and a review of the literature examining Web-based applications used to support practice change in other settings [47,48].

### Statistical Analysis

Descriptive statistics were generated for service characteristics, access, and use of electronic devices. Childcare centers with postcodes ranked in the top 50% of NSW postcodes based on their socioeconomic status (SES) were grouped as being located in *higher socioeconomic areas*, while those in the lower 50% were categorized as being located in *lower socioeconomic areas* using the 2009 Socio-Economic Indexes For Areas (SEIFA), Australia. Childcare centers were categorized as either *rural* (ie, those located in outer regional, remote, and very remote areas) or *urban* (ie, those in regional cities and inner regional areas) based on their postcode using the Accessibility/Remoteness Index of Australia (ARIA).

Similar to that previously used in other studies, TAM subscale scores—perceived usefulness, perceived ease of use, and intention to use—were derived by summing responses—1 to 7—to all items in the subscale and dividing by the number of items within the scale [46]. Descriptive statistics, including mean and standard deviation, and median and interquartile range (IQR), were reported. TAM subscales were dichotomized into a score of 1 (strongly disagree) to 5.9 (slightly agree) or more than or equal to 6 (strongly agree or agree). This cut-point was chosen based on the median score of the subscales and corresponds to those who agree or strongly agree with the items examined within the subscales, providing a clinically meaningful way of interpreting the results.

To examine factors associated with intention to use, a multivariable logistic regression was conducted using the backward stepwise method to exclude variables where *P*>.1. The dependent variable was intention to use, and factors examined were the following: perceived ease of use, perceived usefulness, locality (rural/urban), socioeconomic status (high/low), service type (preschool/nonpreschool), number of children enrolled, number of primary contact staff, number of computers, and whether the center used a childcare management enrolment system software. All significance tests were two-tailed, with an alpha of .05. The proportion and 95% confidence intervals of center managers reporting a system feature as useful/very useful in assisting with the implementation of healthy eating and physical activity-promoting policies and practices were calculated.

## Results

### Service Characteristics

Overall, 277 centers were approached, and 214 (77.3%) consented to participate in the telephone survey. Of the participating centers, 36.9% (79/214) were preschools and 70.6% (151) were long day care centers (see Table 1). Of the centers, 7.5% (16/214) were both preschools and long day care centers. Almost all centers were open for 5 days per week and 77.6% (166/214) were open 8 or more hours per day. Of the centers, 81.8% (175/214) reported being approved under the childcare benefit scheme, and of those, 159/175 (90.9%) reported using a CCMS-approved software program to manage enrolments.

**Table 1:** Descriptive characteristics of participating center-based childcare services (n=214).

Service characteristics	n (%) or mean (SD)
Preschools, n (%)	79 (36.9)
Long day care centers, n (%)	151 (70.6)
Number of children enrolled, mean (SD)	77 (34)
Usual number of primary contact educators, mean (SD)	8.9 (5.2)
Average daily opening hours, mean (SD)	9.6 (1.9)
Open 5 days per week, n (%)	207 (96.7)
Open 8 hours or more per day, n (%)	166 (77.6)
Used CCMS^a^software, n (%)	159 (74.3)

^a^Child Care Management System (CCMS).

### Access and Use of Internet and Associated Equipment

All but two services reported using computers at their center (212/214, 99.1%), with 58.9% (126/214) of all services reporting having access to three or more computers in their center. A total of 40.2% (86/214) of service managers reported using portable tablets in their center. Almost all centers (205/214, 95.8%) had Internet access for at least 1 year and the majority (160/214, 74.8%) reported having Internet access for 5 or more years. More than 90% of service managers reported using computers for administrative and reporting tasks (210/212, 99.1%), to search for information (204/212, 96.2%), and to access emails (206/212, 97.2%), whereas portable tablets were used most frequently to assist with child education (76/86, 88%) (see Table 2).

**Table 2:** Use of computers and portable tablets by childcare center staff.

Tasks	Computer (n=212), n (%, 95% CI)	Portable tablet (n=86), n (%, 95% CI)
Administrative tasks	210 (99.1, 97.9-100)	9 (10, 4-17)
To complete reporting requirements	210 (99.1, 97.8-100)	11 (13, 6-20)
For staff education	193 (91.0, 87.2-94.9)	22 (26, 16-35)
To assist with child education in the classroom	174 (82.1, 76.9-87.2)	76 (88, 82-95)
To search for information	204 (96.2, 93.6-98.8)	42 (49, 38-60)
To access emails	206 (97.2, 94.9-9.49)	12 (14, 7-21)
For planning and recording daily programming of service and children's activities	180 (84.9, 80.1-89.7)	34 (40, 29-50)
To report progress and provide information to parents or staff	187 (88.2, 83.9-92.5)	19 (22, 13-31)
Other^a^	2 (0.9, 0-2.2)	8 (9, 3-16)

^a^Includes use for networking or linking to other services, to play music, as a communication aid for special-needs children, and to display graphs.

### Factors Associated With Intention to Use a Web-Based Program to Implement Policies and Practices

The mean score for intention to use a Web-based program (n=210) was 5.9 (SD 1.5), for perceived usefulness was 5.3 (SD 1.6), and for perceived ease of use was 5.5 (SD 1.1). The median score for intention to use was 6.0 (IQR 5.3-7.0), for perceived usefulness was 5.7 (IQR 4.8-6.8), and for perceived ease of use was 5.7 (IQR 4.8-6.3). Table 3 shows the factors that were associated with intention to use a Web-based program.

Perceived ease of use, perceived usefulness, and number of children enrolled had *P* values less than .1 in the univariate analyses (see Table 3) and were included in the multivariable logistic regression. In the final model, only perceived ease of use (odds ratio [OR] 3.9, 95% CI 1.6-9.2, *P*=.002) and perceived usefulness (OR 28, 95% CI 8.0-95.2, *P*<.001) were significantly associated with a score of more than or equal to 6 for intention to use.

**Table 3:** Factors associated with intention to use a Web-based program to support staff with implementing healthy eating and physical activity policies and practices (n=210).

Factors		Intention to use^a^		Univariate analysis	
		Mean score of 1.0-5.9 (n=59), n (%)	Mean score of 6.0-7.0 (n=151), n (%)	X^2^_1_	*P*
**Locality (n=200)** ^b^				1.2	.30
	Urban	53/56 (95)	130/144 (90.3)		
	Rural	3/56 (5)	14/144 (9.7)		
**Type of childcare center**				1.2	.30
	Preschool	25 (42)	51 (33.8)		
	Nonpreschool	34 (58)	100 (66.2)		
**Use of CCMS** ^c^ **software**				2.4	.10
	Yes	40 (68)	118 (78.1)		
	No/don't know	19 (32)	33 (21.9)		
**Number of children enrolled**				3.5	.06
	75 or less	27 (46)	90 (59.6)		
	>75	33 (54)	60 (39.7)		
**Number of staff members**				1.4	.20
	1 to 8	32 (54)	96 (63.6)		
	>8	27 (46)	55 (36.4)		
**Disadvantage (n=201)** ^b^				2.6	.11
	Low SES^d^	17/56 (30)	62/145 (42.8)		
	High SES	39/56 (70)	83/145 (57.2)		
**Perceived ease of use** ^e^				21.4	<.001
	1.0-5.9	50 (85)	74 (49.0)		
	6.0-7.0	9 (15)	77 (51.0)		
**Perceived usefulness** ^e^				53.1	<.001
	1.0-5.9	56 (95)	58 (38.4)		
	6.0-7.0	3 (5)	93 (61.6)		

^a^Score of 1.0-5.9 indicates response to statements of strongly disagree to slightly agree, and score of 6.0-7.0 indicates response to statements of agree and strongly agree.

^b^Center number is less than the total as no Socio-Economic Indexes For Areas (SEIFA) and Accessibility/Remoteness Index of Australia (ARIA) score matched the center postcode.

^c^Child Care Management System (CCMS).

^d^Socioeconomic status (SES).

^e^Significant variables in multivariable model, *P*<.05.

### Features to Support Healthy Eating and Physical Activity Policy and Practice Implementation

More than 90% of service managers reported the following features to be useful/very useful in supporting the implementation of healthy eating and physical activity policies and practices: decision-support systems to help support staff with planning a healthy menu (117/129, 90.7%), having links to useful nutrition and physical activity resources (212/212, 100%), updated information on nutrition and physical activity guidelines (208/212, 98.1%), and a having a feature which provided updated feedback on how their center was performing in relation to other centers (212/212, 100%) (see Table 4).

**Table 4:** Features of a Web-based program reported by service managers as useful or very useful to help their staff implement healthy eating and physical activity policies and practices (n=212).

Features of Web-based program		n (%)	95% CI
**Performance monitoring and feedback**			
	Allows staff to input nutrition and physical activity information and monitor service’s progress over time.	187 (88.2)	83.9-92.5
	Provides updated information on how your center is performing in relation to other centers.	212 (100)	99.3-100
	Provides staff with tailored feedback based on your service’s needs, and suggested strategies to implement.	198 (93.4)	90.1-96.7
	Allows staff to prioritize which nutrition or physical activity practice they would like to work on.	201 (94.8)	91.8-97.8
**Interaction and communication tools, prompts**			
	Allows staff to ask a member of their local health promotion team for advice.	208 (98.1)	96.2-100
	Allows staff to communicate with staff from other childcare centers via online chat rooms, discussion boards, or blogs.	165 (77.8)	72.2-83.4
	Allows staff to communicate with parents about physical activity and nutrition or via online chat rooms, discussion boards, or blogs.	143 (67.5)	61.5-73.7
	Reminds or prompts staff to deliver a physical activity or nutrition session, based on your service's daily schedule.	182 (85.8)	81.2-90.5
**Provision of education materials**			
	Uses videos or interactive activities, including games, to demonstrate an activity.	194 (91.5)	87.8-95.3
	Includes a database of healthy lunchbox options, which is regularly updated (n=116)^a^.	113 (97.5)	94.5-100
	Provides links to useful physical activity and nutrition resources.	212 (100)	99.3-100
	Provides updated information on nutrition and physical activity guidelines relevant to preschools.	208 (98.1)	96.3-100
Decision-support systems			
	Supports you and your staff in planning a healthy menu for your childcare center (n*=*129)^b^.	117 (90.7)	85.7-95.7

^a^Only services that required parents to provide food for their children answered this question.

^b^Only services that provided food for children answered this question.

## Discussion

### Principal Findings

Almost all childcare center managers had access to computers and the Internet, with 40.2% (86/214) of centers also reporting having access to portable tablets. The majority of service managers reported high behavioral intention to use an electronic Web-based program to support their service with implementing healthy eating and physical activity-promoting policies and practices—71.9% (151/210) of service managers scored an average of 6 or more on intention to use. Constructs within the TAM—perceived ease of use and perceived usefulness—were significantly associated with intention to use. Several preferred features, including the capacity to provide feedback on how the service was performing, providing updated links to physical activity and nutrition resources, and use of decision-support systems to assist with planning menus, were consistently rated as useful or very useful to support practice improvement in this setting. The universal access that childcare center managers have to computers, the high proportion reporting intending to use such systems, and high acceptability of Web-based features suggest that there is considerable potential for electronic programs to be developed to support childcare center staff with implementation of healthy eating and physical activity-promoting policies and practices.

### Comparisons With Prior Work

The near universal access that childcare center managers have to computers and the Internet is not surprising given the introduction of mandatory online recording systems for childcare benefit-approved services in Australia since 2009 [24] . The potential of newer forms of computer technology such as tablets to provide implementation support may increase the appeal of Web-based support programs given their portability, capacity to provide tailored interactive information in multiple formats, ease of navigation, and potential effectiveness in modifying other health behaviors [49].

Consistent with previous research using TAM [40] and other research assessing characteristics associated with uptake of electronic interventions [50], perceived ease of use and perceived usefulness were significantly associated with service managers’ intention to use a Web-based program. Intention to use scores reported in this study are higher than those documented among students [33] and clinicians [40]. While these samples are not directly comparable, such findings suggest greater intention to use electronic Web-based programs among childcare center managers and are encouraging, given findings that behavioral intention to use scores, as measured by TAM, are associated with actual use of the program [44,45]. TAM may be a useful model to inform the design, implementation, and evaluation of electronic Web-based programs in childcare centers.

The provision of training and educational materials, including guidelines and updated links to healthy eating and physical activity resources, were rated as useful by almost all service managers, a finding consistent with other studies which report that childcare center staff find training and resources useful to support their delivery of healthy eating and physical activity policies and practices [51,52]. Communications features which allowed staff to interact with parents and staff from other childcare centers were the least preferred function. This may reflect a preference for more conventional methods—face-to-face, telephone, or one-on-one communication methods—rather than chat rooms or forums as examined in this study. Previous studies in childcare centers have reported using Web-based resources largely for dissemination of information [23,53]. However, these findings suggest that an opportunity exists to use more interactive training resources and decision-support tools to support childcare centers with implementing healthy eating and physical activity-supporting policies and practices.

### Implications

To ensure that the design characteristics of Web-based programs are both functional and easy to use, end-user engagement and feedback on the utility of such interventions need to be undertaken prior to introduction of new electronic interventions [41]. Further examination of the specific design characteristics that are associated with ease of use and perceived usefulness is needed to inform the development of Web-based programs that are most likely to be adopted by childcare center staff. Strategies to increase usefulness, such as incorporating features within the program that deal with tasks currently performed with computers or tablets (eg, reporting or administrative tasks) and engaging end users in development and pilot-testing of the program, could potentially be useful in facilitating uptake [41,45]. Research with clinicians suggest that the provision of staff training, establishing organizational support, and encouraging peer uptake and support may be useful to facilitate uptake of electronic interventions [41,45]. Further descriptive and intervention research examining the association between TAM constructs—perceived ease of use and perceived usefulness—and actual use of an electronic, Web-based program in childcare centers is needed.

### Strengths and Limitations

Strengths of the study include the use of a large, randomly selected sample of centers from across an Australian state and the adaptation of a previously validated tool to assess intention to use a Web-based program. To our knowledge, this is the first study to describe childcare centers’ access to Web-based devices and factors associated with childcare service managers’ intention to use a Web-based program internationally. Nonetheless, a number of study limitations warrant mention. This study assessed intention to use, rather than actual use of electronic Web-based programs. While there is empirical evidence supporting the relationship between intention and actual use [44], and assessments of intention to use provide important formative information for program development [41], rates of actual use are likely to differ from those reported here. Childcare centers within a particular region of NSW—approximately 10% of centers—were also excluded due to participation in another trial [37]. A comparison between excluded centers (n=26) and those in this study found no significant difference in the number of usual providers (*P*=.22) and number of children enrolled (*P*=.88). A significantly larger proportion of centers located in this region were located in lower SES areas (*P*=.003) and rural areas (*P*=.015). As such, findings reported in this study are only likely to be representative of the 90% of centers in NSW from where the centers were randomly sampled. The survey was conducted with service managers of childcare centers who are likely to be involved in overseeing and coordinating the introduction of electronic Web-based interventions in their centers. Future assessments with childcare staff who may be primarily involved in the delivery of healthy eating and physical activity practices are likely to provide useful complementary information on how best to implement such programs in childcare centers. Notwithstanding these limitations, the study provides support for the potential of Web-based technology to make a significant contribution to the translation of evidence-based obesity prevention interventions in this setting.

### Conclusions

Findings reported in this paper highlight the potential for electronic Web-based interventions to be used to support the implementation of healthy eating and physical activity-promoting policies and practices in childcare centers. Further research examining the development and effectiveness of using such modalities to support practice change within childcare centers is warranted to realize the potential of childcare centers for obesity prevention in the community.

## Multimedia Appendix 1

Modified TAM questionnaire (administered via computer-assisted telephone interview).

## Abbreviations

ARIA: Accessibility/Remoteness Index of Australia
CATI: computer-assisted telephone interview
CCMS: Child Care Management System
HMRI: Hunter Medical Research Institute
HNELHD: Hunter New England Local Health District
HNEPH: Hunter New England Population Health
IQR: interquartile range
NHMRC: National Health and Medical Research Council
NSW: New South Wales
OR: odds ratio
SEIFA: Socio-Economic Indexes For Areas
TAM: Technology Acceptance Model
