# Supplementary material for: Childcare Service Centers’ Preferences and Intentions to Use a Web-Based Program to Implement Healthy Eating and Physical Activity Policies and Practices: A Cross-Sectional Study
Source: J Med Internet Res. 2015 Apr 30;17(5):e108. doi: 10.2196/jmir.3639 (PMC4432224; doi:10.2196/jmir.3639)
Supplement: Supplementary file 1 [file jmir_v17i5e108_app1.pdf]

Appendix 1: Modified TAM questionnaire (administered via computer assisted telephone interview)

Information Screen Item

We are interested in finding out how we can use the electronic systems in your service to help support you and your educators deliver healthy eating and physical activity promoting practices. For example, we could include an additional program within electronic systems such as CCMS. This additional program may include features such as links to useful resources, videos or games demonstrating some PA or nutrition learning experiences, and provide current healthy eating and PA recommendations for children.
I'd like to ask your views on using such a program in your service.

Please remember that there are no right or wrong answers - your opinions will help to ensure that such a program would be useful, relevant and practical for use in children's services.

When answering the following questions, we ask you to keep the description of the program in mind. I will now read out a list of statements about the program, and ask you to rate them on a scale of 1 - 7, where:
7 = strongly agree,
1 = strongly disagree, and
4 = neither agree nor disagree.

Perceived usefulness (this heading not included in the questionnaire)

1. I would find this program useful in my service to help staff with delivering nutrition and physical activity policies and practices.

1 Strongly Disagree
2 Disagree
3 Slightly disagree
4 Neither agree nor disagree
5 Slightly agree
6 Agree
7 Strongly Agree
8 Don't know [DO NOT READ]
.R Refused [DO NOT READ]

2. Using this program would improve staff performance in delivering nutrition and physical activity policies and practices.

1 Strongly Disagree
2 Disagree
3 Slightly disagree
4 Neither agree nor disagree
5 Slightly agree
6 Agree
7 Strongly Agree
8 Don't know [DO NOT READ]
.R Refused [DO NOT READ]

3. Using this program would increase staff productivity in delivering nutrition and physical activity policies and practices.

1 Strongly Disagree
2 Disagree
3 Slightly disagree
4 Neither agree nor disagree
5 Slightly agree
6 Agree
7 Strongly Agree
8 Don't know [DO NOT READ]
.R Refused [DO NOT READ]

4. Using this program would help enhance the effectiveness of staff delivery of nutrition and physical activity policies and practices.

1 Strongly Disagree
2 Disagree
3 Slightly disagree
4 Neither agree nor disagree
5 Slightly agree
6 Agree
7 Strongly Agree
8 Don't know [DO NOT READ]
.R Refused [DO NOT READ]

Perceived ease of use (this heading not included in the questionnaire)

5. My interaction with this program would need to be clear and understandable.

1 Strongly Disagree
2 Disagree
3 Slightly disagree
4 Neither agree nor disagree
5 Slightly agree
6 Agree
7 Strongly Agree
8 Don't know [DO NOT READ]
.R Refused [DO NOT READ]

6. Interacting with this program is not likely to require a lot of my mental effort.

1 Strongly Disagree
2 Disagree
3 Slightly disagree
4 Neither agree nor disagree
5 Slightly agree
6 Agree
7 Strongly Agree
8 Don't know [DO NOT READ]
.R Refused [DO NOT READ]

7. I would find such a program easy to use.

1 Strongly Disagree
2 Disagree
3 Slightly disagree
4 Neither agree nor disagree
5 Slightly agree
6 Agree
7 Strongly Agree
8 Don't know [DO NOT READ]
.R Refused [DO NOT READ]

8. I would find it easy to get the program to do what I want it to do.

1 Strongly Disagree
2 Disagree
3 Slightly disagree
4 Neither agree nor disagree
5 Slightly agree
6 Agree
7 Strongly Agree
8 Don't know [DO NOT READ]
.R Refused [DO NOT READ]

Behavioural intentions to use (this heading not included in the questionnaire)

9. Assuming I had access to this program, I intend to use it.

1 1 Strongly Disagree
2 2 Disagree
3 3 Slightly disagree
4 4 Neither agree nor disagree
5 5 Slightly agree
6 6 Agree
7 7 Strongly Agree
8 Don't know [DO NOT READ]
.R Refused [DO NOT READ]

10. Given that I had access to the program, I predict that I would use it.

1 Strongly Disagree
2 Disagree
3 Slightly disagree
4 Neither agree nor disagree
5 Slightly agree
6 Agree
7 Strongly Agree
8 Don't know [DO NOT READ]
.R Refused [DO NOT READ]

If this program was currently available, I would plan to use the program in the next 6 months.

1 Strongly Disagree
2 Disagree
3 Slightly disagree
4 Neither agree nor disagree

5 Slightly agree
6 Agree
7 Strongly Agree
8 Don't know [DO NOT READ]
.R Refused [DO NOT READ]
